# A Cattle Behavior Recognition Method Based on Graph Neural Network Compression on the Edge

**DOI:** 10.3390/ani16030430

**Published:** 2026-01-29

**Authors:** Hongbo Liu, Ping Song, Xiaoping Xin, Yuping Rong, Junyao Gao, Zhuoming Wang, Yinglong Zhang

**Affiliations:** 1 Key Laboratory of Biomimetic Robots and Systems, Ministry of Education, School of Mechatronical Engineering, Beijing Institute of Technology, Beijing 100081, China; 3220235260@bit.edu.cn (H.L.); gaojunyao@bit.edu.cn (J.G.); 3220240170@bit.edu.cn (Z.W.); 3120255233@bit.edu.cn (Y.Z.); 2 National Hulunbuir Grassland Ecosystem Observation and Research Station, Institute of Agricultural Resources and Regional Planning, Chinese Academy of Agricultural Sciences, Beijing 100081, China; xinxiaoping@caas.cn; 3 College of Grassland Science and Technology, China Agricultural University, Beijing 100193, China; rongyuping@cau.edu.cn

**Keywords:** cattle behavior recognition, wearable devices, model compression, embedded machine learning

## Abstract

Cattle behavior is closely related to their health status, and monitoring cattle behavior using intelligent devices can assist herders in achieving precise and scientific livestock management. Current behavior recognition algorithms are typically executed on server platforms, resulting in increased power consumption due to data transmission from edge devices and hindering real-time computation. An edge-based cattle behavior recognition method via Graph Neural Network (GNN) compression is proposed in this paper. Firstly, this paper proposes a wearable device that integrates data acquisition and model inference. This device achieves low-power edge inference function through a high-performance embedded microcontroller. Secondly, a sequential residual model tailored for single-frame data based on Inertial Measurement Unit (IMU) and displacement information is proposed. The model incrementally extracts deep features through two Residual Blocks (Resblocks), enabling effective cattle behavior classification. Finally, a compression method based on GNNs is introduced to adapt edge devices’ limited storage and computational resources. The method adopts GNNs as the backbone of the Actor–Critic model to autonomously search for an optimal pruning strategy under Floating-Point Operations (FLOPs) constraints. The experimental results demonstrate the effectiveness of the proposed method in cattle behavior classification. Moreover, enabling real-time inference on edge devices significantly reduces computational latency and power consumption, thereby highlighting the proposed method’s advantages for low-power, long-term operation.

## Simple Summary

Behavior recognition is essential techniques in modern livestock and poultry farming, supporting precision agriculture and improving production efficiency. This study proposes an edge-based cattle behavior recognition method based on Graph Neural Network (GNN) compression. A Sequence Residual Network (S-ResNet) tailored for single-frame inputs is developed, and a lightweight model is obtained via GNN compression. The resulting model is successfully deployed on a wearable device with edge inference capability. Experimental results show that the compressed model achieves an accuracy of 93.20%, while its power consumption is only 46.8% of that required by cloud inference. The proposed method enables edge-side cattle behavior recognition and effectively extends device endurance, making it suitable for automated monitoring in large-scale free-grazing scenarios.

## 1. Introduction

Understanding cattle behavior is crucial for health monitoring, welfare assessment, and productivity improvement [1]. In small-scale farming, cattle behavior abnormalities can be manually observed based on the farmer’s experience. However, in large-scale scenarios, where cattle are often widely dispersed over vast areas, manual observation and recording of animal behavior are labor-intensive and impractical. Therefore, adopting advanced data analysis technologies in high-tech farming is key to improving livestock production’s efficiency and economic benefits [2].

Researchers typically employ two primary methods for monitoring cattle behavior: fixed and wearable. Fixed devices utilize depth cameras, visual cameras, or audio devices installed in stationary scenes [3,4,5,6,7]. Fixed devices offer limited coverage and are only suitable for indoor cattle farming. Wearable devices integrate various sensors (such as pressure sensors, accelerometers, gyroscopes, magnetometers, and temperature sensors) into embedded systems to acquire behavioral data [8,9,10,11]. Wearable devices are suitable for large-scale free grazing due to their small size and light weight, and are widely used in the field of cattle behavior recognition [12,13,14].

In the research of wearable device behavior recognition algorithms, machine learning has gradually become the mainstream method in behavior classification tasks due to its ability to efficiently extract and describe features [15,16]. Many studies have employed models such as Multilayer Perceptron (MLP) [17], Convolutional Neural Networks (CNNs) [18], Recurrent Neural Networks (RNNs) [19], and Long Short-Term Memory (LSTM) [20,21] networks for behavior monitoring. However, in most of these studies, the wearable devices are primarily used for data acquisition, while model inference is largely performed on the servers or in the cloud. This latency inference requires storing sensor data on the device or transmitting raw data through wireless communication. Beyond low recognition efficiency, wireless transmission of raw data poses significant challenges to the battery life of wearable devices, where frequent battery replacements compromise monitoring continuity [22].

To overcome the latency and power consumption bottlenecks caused by cloud inference, an increasing number of studies have adopted lightweight models and efficient inference frameworks to move the inference process from servers to edge devices [23,24]. Recently, tiny machine learning techniques have gained traction in edge computing and embedded devices, aiming to enhance inference speed and energy efficiency by leveraging lightweight models and model compression techniques [25,26]. Among these techniques, pruning is a key strategy that reduces redundant parameters and computational complexity. Various studies have proposed different pruning strategies and optimization approaches to achieve optimal model performance, including methods based on norms [27], magnitudes [28], sparse regularization [29], loss change [30], reinforcement learning [31], and meta-learning [32]. Recently, there has been a growing trend of extending Automated Machine Learning (AutoML) methods and Neural Architecture Search (NAS) to pruning [33].

In recent years, graph neural networks (GNNs) have also been increasingly introduced into model compression and acceleration. Unlike approaches that treat net-work layers as independent features, GNNs can model the layers or channels of a neural network as a graph, thereby facilitating the extraction of structural dependencies across layers and their respective contributions to overall performance and computational overhead. Prior studies have noted both data redundancy and computational redundancy in two fundamental GNN operations, namely propagation and transformation. Accordingly, hierarchical compression frameworks have been proposed to re-duce redundancy and accelerate training and inference by compressing graph structures and exploiting similarities among node features [34]. Beyond model acceleration, GNNs have also been applied to coding and transmission scenarios. For multi-view data such as light-field video, GNN-based compression and multiple-description transmission strategies have been developed to jointly optimize compression efficiency and reconstruction quality under unstable network conditions [35]. In large-scale spatiotemporal data analytics for smart cities, GNNs have been employed for predictive compression, improving compression efficiency and controllability while maintaining decompression accuracy [36]. However, most compressed models rely on sparse matrix operations and hardware acceleration, which are unsuitable for extremely resource-constrained wearable devices. Furthermore, wearable devices have their own recognition tasks and are unsuitable for collaborative distributed inference methods [37].

In summary, fixed monitoring systems are constrained by deployment conditions, resulting in limited coverage and scalability, which makes them difficult to apply in large-scale free-range grazing environments. Although wearable devices are better suited for monitoring in open pastures, their limited on-device computing resources constrain edge inference. As a result, existing approaches often rely on wireless transmission or offline data transfer to servers for inference, thereby limiting real-time monitoring capability. A substantial knowledge gap remains in current technologies. On the one hand, for continuous multi-source time-series data in grazing scenarios, such as IMU and GPS data, there is still a lack of lightweight model designs that can perform on-device inference directly on the edge. On the other hand, a general-purpose compression framework that does not rely on specialized hardware is still missing, such that the compressed models can preserve recognition accuracy while simultaneously reducing computational overhead. To address these challenges, this paper proposes a cattle behavior recognition method based on Graph Neural Networks (GNNs) on the edge. The main contributions are as follows:

- Develop a wearable device with model inference capability: The device utilizes an embedded microcontroller to provide hardware support for model inference, transferring the model inference workload from the cloud to the edge.
- Construct a Sequence Residual Network (S-ResNet) model: The model is suitable for single-frame data containing time-series information and can effectively reduce the storage and computation of edge data.
- Propose a compression method based on GNNs: The method employs GNNs as the foundational network for the Actor–Critic model. Graph-structured data was constructed, and the TD3 algorithm was introduced to update the reinforcement learning strategy. The resulting lightweight model does not rely on hardware acceleration and is well-suited for deployment on edge devices.
- Deploy the S-ResNet model on wearable devices: Different variants of the S-ResNet model are deployed on wearable devices and compared with cloud inference for power consumption.

## 2. Materials and Methods

### 2.1. Wearable Devices

**Figure 1:**
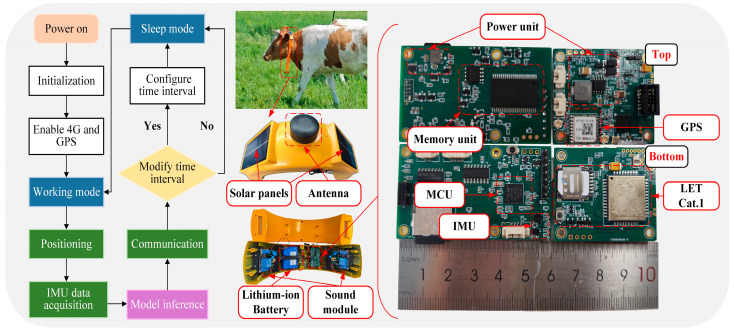
The internal composition and workflow of the wearable device.

The wearable device developed in this study is illustrated in Figure 1. It comprises a lithium-ion battery, solar panels, an antenna, a processing module, and a sound module (the sound module is not discussed in this paper and is intended for executing behavior-triggered actions after cattle behavior recognition). The processing module includes a Microcontroller Unit (MCU), an Inertial Measurement Unit (IMU), a power unit, a storage unit, a Global Positioning System (GPS), and a communication module. After the device is powered on, it first performs initialization and enables the communication and positioning chips. It then enters the working mode and starts localization as well as IMU data acquisition. Model inference is conducted based on the collected data (inference is not required if the device is used only for data acquisition). The data are transmitted to the cloud, and the device receives commands from the cloud indicating whether the working time interval should be reconfigured. If reconfiguration is required, the device updates the settings and then enters sleep mode; otherwise, it enters sleep mode directly. The sleep mode helps extend the device’s endurance.

The device employs the STM32U585 microcontroller from STMicroelectronics (Geneva, Switzerland), featuring an ultra-low-power 32-bit Arm Cortex-M33 CPU with 2 MB of Flash memory and 786 KB of Static Random-Access Memory (SRAM). It uses the LYNQ N10 module from Shanghai Mobiletek Communication (Shanghai, China) for positioning and the ML307A-GCLN Long Term Evolution Category 1 (LTE Cat.1) module from China Mobile Internet of Things (Chongqing, China) for 4G data transmission. The IMU sensors are the STMicroelectronics (Geneva, Switzerland) ISM330DHCX and IIS2MDC. The ISM330DHCX integrates a 3-axis digital accelerometer and a 3-axis digital gyroscope, while the IIS2MDC is a digital magnetic sensor.

The wearable device is powered by a 3.7 V, 9000 mAh lithium-ion battery, which can be recharged via two solar panels mounted externally on the casing. Under optimal sunlight conditions, the solar panels provide a charging current of approximately 130 mA when the device is placed vertically and about 200 mA when oriented directly toward the sun. On cloudy days, the charging current is reduced to around 13 mA.

### 2.2. Dataset

#### 2.2.1. Dataset Acquisition

**Figure 2:**
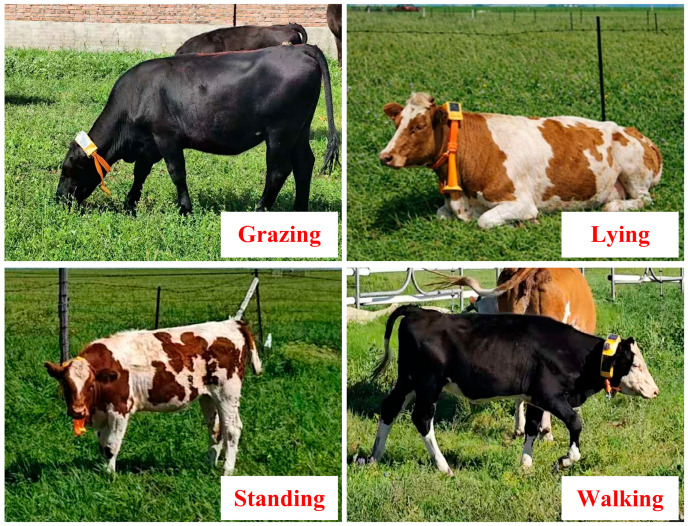
Four-class cattle behaviors.

The experiment was conducted at the National Hulunbuir Grassland Ecosystem Observation and Research Station (49.32–49.34° N, 119.94–119.96° E, 670–677 m a.s.l.) in Inner Mongolia, China. The region is characterized by a temperate semi-arid inland climate with a mean temperature of 22 to 27 °C. The experiment was divided into two phases: the first phase was conducted during August-September 2024, and the second phase was conducted during July–August 2025. The livestock are Simmental (red and white) cattle and Angus cattle. A total of 20 adult cattle (about 500 kg) were selected to wear the device for monitoring and observation. The devices were placed on the cattle’s neck and secured them with a strap and a counterweight, as illustrated in Figure 2. During the experiment, the cattle were allowed to feed freely, while ensuring the devices remained securely attached and no signal interruptions occurred. In addition, the device adopts a waterproof, sealed design to ensure stable operation under field conditions such as rainfall and high humidity. During data collection, daily environmental information and communication link status are recorded synchronously to support data archiving under different environmental conditions. When short-term network fluctuations occur, the data are temporarily buffered on the device and retransmitted after the signal is restored, thereby ensuring data integrity.

All sensor data were transmitted to cloud storage during the data acquisition phase. Following model deployment on edge devices, data processing occurred locally on the edge. The wearable device synchronously collected IMU and GPS data under a common clock source and transmitted them to the cloud via 4G communication for storage. Each data packet was assigned a unique identifier and linked to the corresponding cattle, ensuring accurate mapping between behavioral data and individual animals. The cattle behaviors were annotated through visual observation, with the categories defined as shown in Figure 2 and Table 1. To reduce potential bias arising from subjective differences, the annotations were independently completed by two observers following consistent criteria. Inter-observer agreement was quantitatively evaluated using Cohen’s Kappa coefficient to characterize the reliability of the annotations [38].

#### 2.2.2. Dataset Construction and Processing

The single-frame data containing time-series information was constructed using GPS displacement and IMU pose features. Each data frame contained ten features: triaxial acceleration, triaxial gyroscope, triaxial magnetometer data, and displacement d [39]. The displacement feature represented the movement distance calculated from two GPS positions recorded at a 10 s interval. The IMU sensors sampled at 1 Hz, and the average values over the sampling period were used as feature representations.

(1) $$d=2r\arcsin\left(\sqrt{\sin^{2}\left(\frac{lat_{2}-lat_{1}}{2}\right)+\cos\left(lat_{1}\right)\cos\left(lat_{2}\right)\sin^{2}\left(\frac{lon_{2}-lon_{1}}{2}\right)}\right)$$

where $lat_{1}$ and $lat_{2}$ represent the latitudes of the two positions, $lon_{1}$ and $lon_{2}$ represent the longitudes of the two positions, and r is the Earth’s radius, approximately 6371.393 km.

Due to the limited number of collected samples, potential sampling bias may exist and the class distribution is imbalanced. To mitigate the impact of class imbalance on prediction performance and improve the model’s generalization ability, Synthetic Minority Over-sampling Technique (SMOTE) was applied to oversample the minority classes for data augmentation [40,41]. In dataset D, class $D_{c}$ was identified as a minority class, with the corresponding samples denoted as $x\in D_{c}$. The synthetic samples were generated as follows:

(2) $$x_{new}=x+\varsigma \cdot \left(x_{NN}-x\right),\varsigma ∼u(0,1)$$

where $x_{NN}$ represents a randomly selected sample from the k-nearest neighbors, $x_{NN}\in D_{c}$, and ς follows a uniform distribution.

The augmented dataset was randomly partitioned into training and testing sets with a 7:3 ratio. Ten types of feature data were standardized using the mean and standard deviation calculated from the training set. Here, $n^{i}$ denotes the number of samples for the *i*-th type of feature, and $x^{i}$ represents the *i*-th type of feature data, i=1,⋯,10.

(3) $$\mu^{i}=\frac{1}{n^{i}}\sum_{j=1}^{n^{i}}x_{j}^{i},\;\sigma^{i}=\sqrt{\frac{1}{n^{i}}\sum_{j=1}^{n^{i}}{\left(x_{j}^{i}-\mu^{i}\right)}^{2}},x_{std}^{i}=\frac{x^{i}-\mu^{i}}{\sigma^{i}}$$

where $\mu^{i}$ is the mean of the *i*-th type of feature data, $\sigma^{i}$ is the standard deviation of the *i*-th type of feature data, and $x_{std}^{i}$ is the normalized data of the *i*-th type of feature data.

### 2.3. Proposed Method for Cattle Behavior Recognition

#### 2.3.1. Method Overview

**Figure 3:**
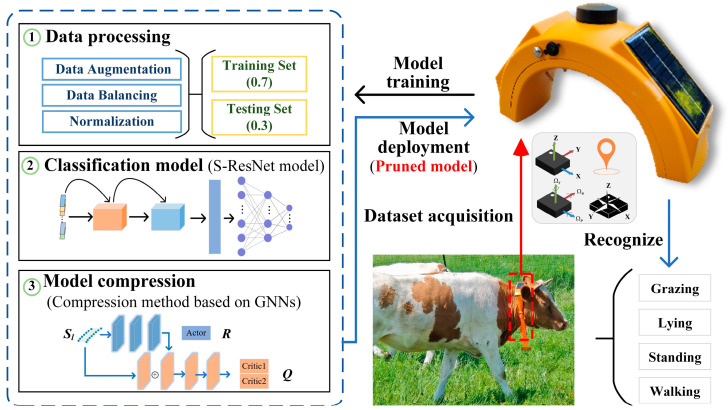
Method overview.

This study recognizes cattle behavior using pose and positional data collected from wearable devices equipped with built-in IMU and GPS. The trained classification model is deployed on edge wearable devices, leveraging their embedded systems for real-time inference. As illustrated in Figure 3, cattle behavior recognition on the edge is divided into two stages: model training and model inference. Data processing, classification model training, and model compression are performed on a personal computer during the training phase to obtain a lightweight model. In the inference phase, the lightweight model is deployed on the wearable device, which collects real-time data as input to generate behavioral predictions.

Neural network compression techniques are utilized to reduce the memory footprint and computational complexity of classification model inference while maintaining model accuracy as much as possible. Suppose the neural network model contains *L* prunable layers and the retention rate *R* is defined as $R\in \left[0,1\right]$. The optimization goal is to maximize the accuracy of the neural network model *M* under the constraint of *R*, expressed as:

(4) $$\begin{array}{l} \underset{{\left\{R_{L}\right\}}_{l=1}^{L}}{\max}accuracy(M_{{\left\{R_{L}\right\}}_{l=1}^{L}}(D))\\ s.t.\frac{FLOPs_{pruned}}{FLOPs_{original}}\leq R \end{array}$$

where D denotes the dataset utilized by the neural network model, $R_{l}$ denotes the retention rate of the *l*-th layer, and Floating-Point Operations (FLOPs) represent the required number of floating-point operations.

#### 2.3.2. S-ResNet Model

The model input consists of statistical features extracted from the raw IMU signals within a fixed time window, together with the corresponding displacement. Although each sample is fed into the network in a single-frame form, the features implicitly encode motion sequence information over the time interval. To handle this one-dimensional feature representation with an inherent sequential structure, the S-ResNet is developed. Using one-dimensional convolutions and residual connections, the sequential features are modeled hierarchically, which significantly reduces computational complexity while maintaining classification accuracy, thereby meeting the requirements for edge deployment on wearable devices.

**Figure 4:**
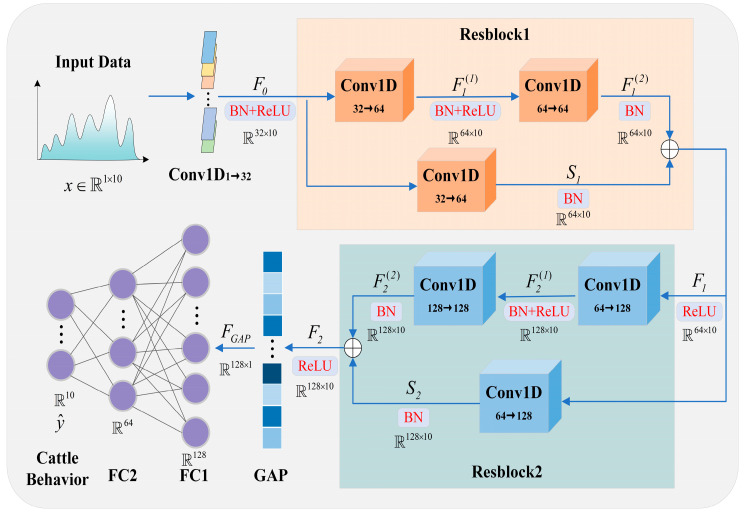
S-ResNet model. (The arrow indicates the direction of data transmission, with Resblock1 on an orange background and Resblock2 on a blue background.)

The architecture of the S-ResNet model is illustrated in Figure 4. The model input data $x\in {\mathbb{R}}^{1\times 10}$ corresponds to the mean values of tri-axis accelerometer, tri-axis gyroscope, tri-axis magnetometer, and displacement, directly matching the raw data format of the sensors. The initial convolutional layer that maps the single-channel input into 32 feature channels. This convolutional operation captures the interrelationships among data from multiple sensors, such as the coupling between acceleration and angular velocity.

(5) $$F_{0}=ReLU(BN(Conv1D_{1\to 32}(x)))$$

where $F_{0}$ denotes the feature map obtained from the first convolutional layer, where the convolution kernel size is 3. BN represents a batch normalization operation, Rectified Linear Unit (ReLU) is the activation function.

The model progressively extracts deeper-level features through two Residual Blocks (Resblocks). Each Resblock consists of two convolutional layers and a shortcut connection, which helps maintain gradient stability and preserve feature integrity. All convolutional layers use a kernel size of 3, except those in the shortcut connections, which use a kernel size of 1. Batch normalization is incorporated to ensure training stability, while the ReLU provides nonlinear enhancement.

(6) $$F_{1}^{\left(1\right)}=ReLU(BN(Conv1D_{32\to 64}(F_{0})))$$

(7) $$F_{1}^{\left(2\right)}=BN(Conv1D_{64\to 64}(F_{1}^{\left(1\right)}))$$

where $F_{1}^{\left(1\right)}$ and $F_{1}^{\left(2\right)}$ represent the output feature maps of the two convolutional layers in Resblock1, respectively. The convolution operations further integrate high-order relationships across channels, such as the orientation correlation between the magnetometer and gyroscope. The two convolutional layers expand the number of channels to 64, gradually extracting abstract features from the signal.

(8) $$S_{1}=BN(Conv1D_{32\to 64}(F_{0}))$$

(9) $$F_{1}=ReLU(F_{1}^{\left(2\right)}+S_{1})$$

where $S_{1}$ is obtained by dimension expansion to match the main branch in size, ensuring element-wise addition can be performed. The shortcut connection helps mitigate the degradation problem in deep networks, enhances gradient flow, and prevents information loss in deeper layers. $F_{1}$ represents the output feature map of ResBlock1.

The Resblock2 expands the channels to 128, enabling the model to capture complex behavioral patterns and distinguish subtle differences between similar behaviors (such as Standing and Grazing).

(10) $$F_{2}^{\left(1\right)}=ReLU(BN(Conv1D_{64\to 128}(F_{1})))$$

(11) $$F_{2}^{\left(2\right)}=BN(Conv1D_{128\to 128}(F_{2}^{\left(1\right)}))$$

(12) $$S_{2}=BN(Conv1D_{64\to 128}(F_{1}))$$

(13) $$F_{2}=ReLU(F_{2}^{\left(2\right)}+S_{2})$$

where $F_{2}^{\left(1\right)}$ and $F_{2}^{\left(2\right)}$ represent the output feature maps of the two convolutional layers in Resblock1, respectively. $S_{2}$ is obtained by increasing the dimension of $F_{1}$ to 128. $F_{2}$ represents the output feature map of ResBlock2.

The model employs a Global Average Pooling (GAP) layer to compress sequential dimensions, followed by two Fully Connected (FC) layers for the final classification output.

(14) $$F_{GAP}=GAP(F_{2})$$

(15) $$\hat{y}=FC2(FC1(F_{GAP}))$$

where $\hat{y}$ represents the predicted probability of each category. *FC*1 reduces the dimension to 64 to obtain a more compact intermediate representation, and *FC*2 reduces the dimension to the number of categories.

Single-frame data containing time-series information has both temporal dynamic characteristics and avoids high computational cost sliding windows. Small-size convolution kernels and residual structures achieve multimodal fusion while balancing accuracy and efficiency. The S-ResNet model, with its lightweight design, performs cattle behavior recognition efficiently on embedded systems.

#### 2.3.3. Compression Method Based on GNNs

**Figure 5:**
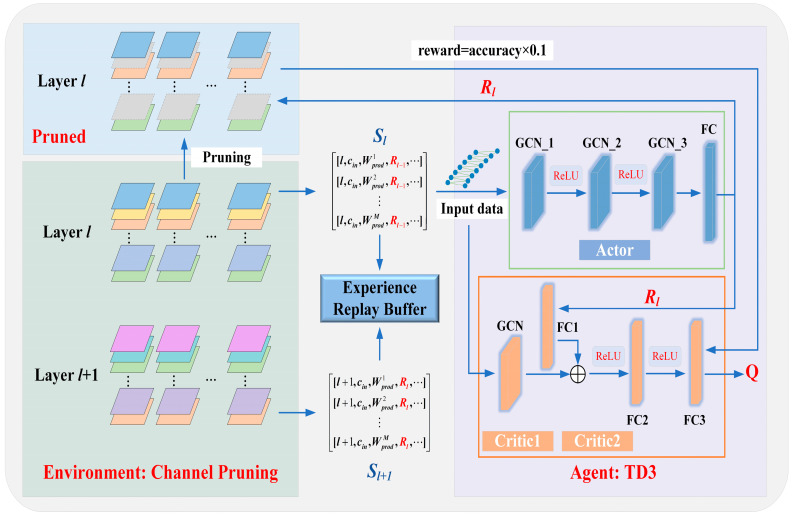
Compression method based on GNNs. (The arrow indicates the direction of data transmission. The green background represents the Environment. The purple background is Agent. The blue background is the pruned model.)

To further reduce the resource consumption of the model on edge devices, this paper proposes a model compression method based on GNNs, as shown in Figure 5. This compression method is framed within a reinforcement learning approach, using Twin Delayed Deep Deterministic Policy Gradient (TD3) for policy updates. GNNs are the backbone for both the Actor and Critic networks in this method. The Actor network consists of three Graph Convolutional Network (GCN) layers with intermediate output dimensions of 400 and 300, respectively. A sigmoid activation function is applied to constrain the retention rate within the range of $\left[0,1\right]$. The two Critic networks have identical architectures. Each Critic begins with a graph convolutional layer that processes the node feature graph, followed by a fully connected layer that processes the action features. These features are then mapped to the same dimensionality of 400. The combined graph and action features are passed through two fully connected layers to compute the reward, with an intermediate dimension of 300.

First, the trained neural network model features are selected as the state space for reinforcement learning. Given the high sensitivity of model compression to retention rates, the action space is defined as a continuous range *R*, where $R\in \left[0,1\right]$. This approach enables more precise compression compared to using discrete actions. The TD3 algorithm is employed as the reinforcement learning agent. The agent receives the state $S_{l}$ from the environment and determines the retention rate $R_{l}$ for the *l*-th layer. It then transitions to the next state $S_{l+1}$ provided by the environment. This iterative process continues until the final layer is processed. The reward value *reward* is subsequently calculated and stored in the experience replay buffer. This study determines the pruning strategy using a two-stage procedure consisting of exploration and exploitation. During the exploration stage, to sufficiently cover the strategy space and enhance the diversity of collected experience, the agent samples actions by taking the deterministic output of the Actor as the mean and adding truncated Gaussian noise. The noise scale decays exponentially with training episodes, enabling a smooth transition from exploration to exploitation. In the environment (channel pruning), a structured channel-pruning policy is adopted to execute the actions. Specifically, for layer *l*, the weight tensor associated with each input channel is treated as a pruning unit, and its L2-norm is computed as the channel-importance metric. The channels are then ranked in descending order of importance, and the top $R_{l}\times c_{in}$ channels are retained. The remaining channels are removed along with their associated convolution kernels and connection weights in the subsequent layers, resulting in a pruned model.

The construction of graph-structured data is critical for the proposed compression method. Graph-structured data consists of nodes and edges representing the complex relationships among nodes. GNNs learn the features of each node by propagating and aggregating information from neighboring nodes, enabling information exchange between nodes [42]. In the context of compression tasks, a graph structure based on model channels is constructed as the state space, where each channel is treated as a node, and edges represent dependency relationships between closely related channels. Each layer of the network model corresponds to a graph structure, with the state space $S_{l}$ for the *l*-th layer defined as follows:

(16) $$\left[\begin{array}{c} \left[l,t,c_{in},c_{out},stride,k,W_{prod}^{1},W_{norm}^{1},reduced_{l},rest_{l},R_{l-1}\right]\\ \vdots \\ \left[l,t,c_{in},c_{out},stride,k,W_{prod}^{m},W_{norm}^{m},reduced_{l},rest_{l},R_{l-1}\right]\\ \vdots \\ \left[l,t,c_{in},c_{out},stride,k,W_{prod}^{M},W_{norm}^{M},reduced_{l},rest_{l},R_{l-1}\right] \end{array}\right]$$

where *l* represents the layer index, and *t* represents the layer type. The weight dimensions are $c_{out}\times c_{in}\times k\times k$, and $c_{in}=M$. The *stride* refers to the step size, $W_{prod}^{m}$ is the product of parameters for the *m*-th input channel in the *l*-th layer, and $W_{norm}^{m}$ represents the L2-norm of the parameters for the *m*-th input channel in the *l*-th layer. The $reduced_{l}$ denotes the total FLOPs reduced by the preceding layers, while $rest_{l}$ represents the number of remaining FLOPs in subsequent layers. $R_{l-1}$ indicates the retention rate of the previous layer [31].

This study models layer-wise channel pruning as a Markov decision process. For layer *l*, the state $S_{l}$ is represented as graph-structured data, where nodes denote channels and edges capture channel dependencies. The Actor takes $S_{l}$ as input and outputs a continuous action $R_{l}$, which is mapped to [0, 1] via a sigmoid function. The reward is calculated only after pruning the final layer, defined as reward=accuracy×0.1, while the reward is set to 0 for all other layers.

The TD3 algorithm obtains a continuous action space in the policy update process, as shown in Algorithm 1. Compared to the Deep Deterministic Policy Gradient (DDPG), TD3 introduces two Critic networks to compute two Q-values, ultimately selecting the smaller Q-value as the target [43]. This clipped double Q-learning structure mitigates the risk of overestimating Q-values, enhancing the algorithm’s stability in noisy environments. When updating the target Critic network, a small Gaussian noise term is added to the action R output by the Actor to perform smoothing, which stabilizes the computation of the target Q-value. This perturbation prevents overly deterministic policies from leading to suboptimal local solutions. The discount factor *γ* balances the trade-off between immediate and future rewards. Meanwhile, the Actor network is updated in a delayed manner and is maintained via an exponential moving average controlled by the soft-update coefficient τ. This design avoids policy instability caused by overly frequent updates.

**Algorithm 1**: TD3 algorithm strategy update**Input**: Original model M, Dataset D, $T,\;\;T_{1},\;\;\gamma ,\;\;\delta_{1},\;\;\delta_{2},\;\;a,\;\;b$**Output:** Pruning strategy *R*

$\mathrm{Initialize}\;Critic_{\theta_{1}},Critic_{\theta_{2}},Actor_{\phi }$


$\;\mathrm{with}\;\mathrm{random}\;\mathrm{parameters}\;\theta_{1},\;\theta_{2},\;\phi$



$\mathrm{Initialize}\;\mathrm{target}\;\mathrm{networks}\;\theta_{1}^{\prime }\leftarrow \theta_{1},\;\theta_{2}^{\prime }\leftarrow \theta_{2},\;\phi^{\prime }\leftarrow \phi$

**Initialize** *S* according to the graph structure data formatfor t=1:T **do**         **for** *l* in *L* **do**                  $R_{l}\leftarrow Actor_{\phi }(S_{l})+\epsilon_{1}$
$\;\mathrm{if}\;t>T_{1}$
$\;\mathrm{else}\;R_{l}\leftarrow Random(a,b)$
                  $\;\epsilon_{1}\sim TN(a,b,R_{l},\delta_{1})$
                  $M^{\prime }\leftarrow$ Prune the input channel of the *l*-th layer of model M based on L2-norm                  $r_{l}=Acc_{M^{\prime }}(D)\times 0.01$ **if** *l*==*L* **else** 0                  $\mathrm{Update}\;reduced_{l+1},\;rest_{l+1},\;R_{l}$
$\;\mathrm{in}\;S_{l+1}$
                  $\mathrm{Store}\;\mathrm{transition}\;\mathrm{tuple}\;(S_{l},R_{l},r_{l},S_{l+1})$ in replay buffer
B
                  **if**
*l*==*L* and $t>T_{1}$
**then**                        $\mathrm{Sample}\;\mathrm{mini-batch}\;\mathrm{of}\;N\;\mathrm{transitions}\;(S,R,r,S^{\prime })$ from B
                        $\tilde{R}\leftarrow Actor_{\phi^{\prime }}(S^{\prime })+\epsilon_{2},\;\epsilon_{2}\sim N(0,\delta_{2})$
                        $y\leftarrow r+\gamma min_{i=1,2}Critic_{\theta_{i^{\prime }}}(S^{\prime },\tilde{R})$
                        $\mathrm{Update}\;\mathrm{Critics}\;\theta_{i}\leftarrow argmin_{\theta_{i}}N^{-1}\sum (y-Critic_{\theta_{i}}(S,R){)}^{2}$
                        if tmod2 **then**                                Update *ϕ* by the deterministic policy gradient:                                $\nabla_{\phi }J(\phi )=N^{-1}\sum \nabla_{a}Critic_{\theta_{1}}(S,R){|}_{a=Actor_{\phi }(S)}\nabla_{\phi }Actor_{\phi }(S)$
                                Update target networks:                                $\theta_{i^{\prime }}\leftarrow \tau \theta_{i}+(1-\tau )\theta_{i^{\prime }}$
                                $\phi^{\prime }\leftarrow \tau \phi +(1-\tau )\phi^{\prime }$
                        **end**
               **end**
      **end**

**end**


Pruning operations on the original model are performed based on the optimal policy. Compared to fine-grained pruning, which operates at the weight level, coarse-grained pruning directly targets the channel level. For example, the weight dimensions of the *l*-th layer are $c_{out}\times c_{in}\times k\times k$, the weight tensor is compressed to $c_{out}\times c_{in}^{\prime }\times k\times k$. In this case, the sparsity of the *l*-th layer is given by $c_{in}^{\prime }/c_{in}$, and the retention rate is defined as $R_{l}=1-c_{in}^{\prime }/c_{in}$.

Graph convolution aggregates neighborhood information and can automatically extract channel topological relationships, overcoming the limitation of conventional pruning methods that ignore complex inter-channel dependencies. Compared to DDPG, TD3 has stronger convergence in noisy environments and is suitable for optimizing high-dimensional continuous action spaces for model compression. Compared to rule-based methods, this approach utilizes reinforcement learning to reduce dependence on manual rule design. Compared to sparse regularization, this method directly optimizes for model accuracy and computational efficiency, balancing pruning rate and performance loss to achieve the optimal pruning strategy. Compared to loss-based compression and meta-learning, the TD3 algorithm updates using experience replay to eliminate the need for multiple forward passes to compute loss changes, thus reducing computational complexity. Compared to fine-grained pruning, coarse-grained pruning reduces hardware adaptation difficulty and does not rely on specific hardware architectures. Therefore, the model pruned with coarse-grained methods can be deployed on any Internet of Thing (IoT) edge device, including wearable devices [44].

## 3. Results

### 3.1. Experimental Environment Configuration and Hyperparameters Settings

The S-ResNet model training and compression strategy search were conducted using PyTorch 1.7.1 and Python 3.7.3 on an NVIDIA Tesla T4 GPU. The specific parameters for model training are shown in Table 2. The fine-tuning parameters for the model after pruning were kept identical. The parameters of the model compression are shown in Table 3.

The Adam optimizer was selected for model training. To balance convergence speed and training stability, the learning rate was set to 0.001. To avoid overfitting during training, the training epochs were set to 100. Considering the memory capacity of the Tesla T4 GPU, the batch size was set to 128. This paper adopted the cross-entropy loss function commonly used in classification tasks [45]. During the model compression strategy search, a smaller batch size of 64 was used to reduce per-step memory consumption. Exploring with 100 episodes can establish an effective initial experience buffer. The compression strategy gradually converged to an optimal solution after 500 episodes. To prevent the loss of essential features caused by excessive pruning, the minimum channel retention rate for each layer was set to 0.2. The soft update coefficient was set to 0.01 to ensure the target network’s stability and learning efficiency.

### 3.2. Evaluation Indicators

In order to verify the performance of the model, accuracy, sensitivity and precision were used to evaluate the performance of the model. Its calculation formula is as follows:

(17) $$accuracy=\frac{TP+TN}{FP+FN+TP+TN}$$

(18) $$sensitivity=\frac{TP}{TP+FN}$$

(19) $$precision=\frac{TP}{TP+FP}$$

where *TP*, *TN*, *FP* and *FN* are the number of true positive cases, true negative cases, false positive cases and false negative cases.

This study reported accuracy, sensitivity, and precision as the average values from five repeated randomized experiments.

### 3.3. Result of Cattle Behavior Recognition

**Figure 6:**
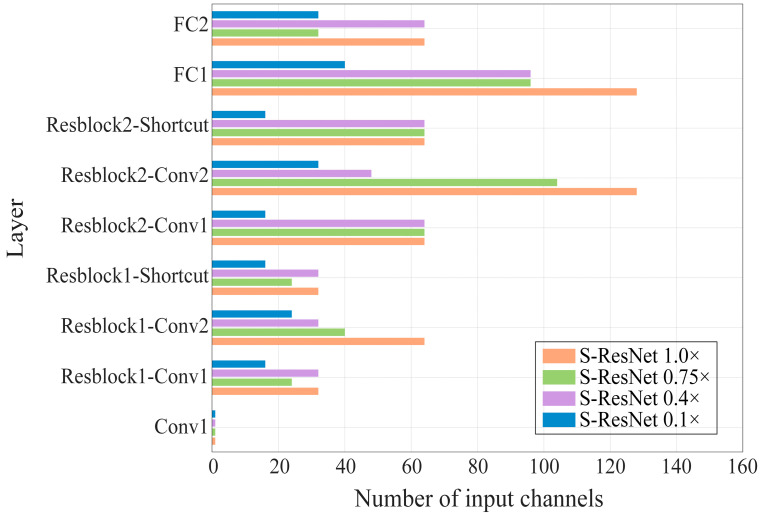
The number of input channels per layer for different S-ResNet model variants.

Based on the experimental settings described in Section 3.1, the S-ResNet 1.0× model was trained on the dataset introduced in Section 2.2. The model was then pruned using the compression method based on GNNs, followed by fine-tuning to obtain different S-ResNet model variants. The number of input channels for each layer of the S-ResNet model variants is shown in Figure 6. Conv1, as the first layer, determined its input channel count based on the dimensions of the input data and was excluded from pruning. The shortcut layers were required to maintain the same number of channels as the main convolutional layers. As the channel retention rate decreased, the pruning strategy prioritized reducing the number of channels in less critical layers. Consequently, the overall channel count of the model exhibited a non-uniform yet significant reduction.

**Figure 7:**
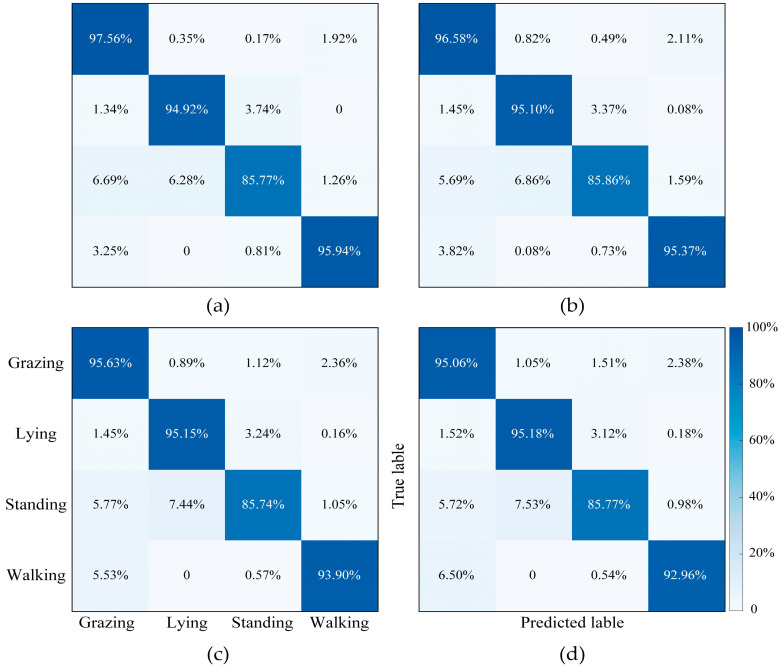
Confusion matrix. (**a**) S-ResNet 1.0× model; (**b**) S-ResNet 0.75× model; (**c**) S-ResNet 0.4× model; (**d**) S-ResNet 0.1× model.

The classification accuracies of S-ResNet 1.0×, ResNet 0.75×, ResNet 0.4×, and S-ResNet 0.1× are 94.50% (±0.09%), 94.09% (±0.11%), 93.53% (±0.10%), and 93.20% (±0.18%), respectively. The confusion matrices of each model are shown in Figure 7. A confusion matrix is a 2-D square matrix, where one dimension contains actual classes (True label), and the other represents the classes predicted by the model (Predicted label). In the figure, the sensitivity for each behavior is explicitly displayed. The sensitivities for Grazing, Lying, and Walking behaviors exceed 90%, whereas the sensitivity for Standing is slightly above 85%. The Standing behavior is more likely to be misclassified as Grazing or Lying. These results demonstrate that the proposed model can effectively recognize cattle behaviors and maintain good performance even after pruning.

### 3.4. Deployment and Inference on the Edge

The S-ResNet model was deployed on a wearable device using STM32Cube.AI to enable real-time recognition of cattle behavior. STM32Cube.AI is a tool and framework provided by STMicroelectronics for running AI models on STM32 microcontrollers [46]. It converts deep learning models into efficient code optimized for STM32 microcontrollers, enabling embedded devices to perform artificial intelligence tasks efficiently. Meanwhile, STM32Cube.AI itself supports model compression.

Table 4 presents computational complexity, memory usage, and inference time when executing model inference on the wearable device. Multiply–accumulate (MACC) represents the total number of multiplication-accumulation operations the network requires. Flash memory includes the model weights, inference engine, and necessary functions. Random Access Memory (RAM) usage encompasses activation values during model inference, input and output data, and other temporary data. Time is required to infer a single data frame, excluding data acquisition time. The MCU in the wearable device provides access to 2 MB Flash and 768 KB RAM, which are sufficient to meet the memory requirements for model inference. Compared with the S-ResNet 1.0× model, the S-ResNet 0.1× model reduces of 80–90% in MACC, Flash, and Time metrics. Due to unchanged input and output data, the reduction in RAM usage is relatively minor, approximately 47%. When the compression level in STM32Cube.AI was set to High during deployment, only the Flash footprint was reduced, whereas the MACC, RAM usage, and inference time remained unchanged. This is because the adopted compression scheme is based on a k-means clustering method, which is mainly applicable to fully connected layers. For models dominated by convolutional layers, this approach provides limited compression benefits and may adversely affect performance.

The power consumption statistics of the edge inference and conventional cloud inference proposed in this paper were conducted on wearable devices, as shown in Table 5. The device was configured with a sleep mode to reduce power consumption. After each cycle, the device woke up to perform a single behavior recognition task and then returned to sleep mode. For cloud inference, each recognition task required data transmission to and from the cloud, whereas edge inference eliminated the need for data transmission.

Upon waking from sleep mode, the GPS required approximately 30 s to reacquire satellite information and complete positioning, while the 4G module took about 20 s to re-register with the base station and establish communication with the cloud. For a 5 min operational cycle, the power consumption for a single edge inference was 0.388 mAh, approximately 46.8% of the power consumption required for cloud inference (0.829 mAh). Calculations show that the daily power consumption for the edge inference approach is approximately 112 mAh. The device can maintain a balanced power supply by ensuring 30 min of adequate sunlight exposure for daily charging. After deploying the S-ResNet model on the edge device, the classification model consistently provided stable real-time predictions of cattle behavior.

## 4. Discussion

### 4.1. Generalization Experiment of S-ResNet Model

To further demonstrate the classification capability of the S-ResNet model, comparative experiments were conducted on a public dataset against other methods. The dataset also includes ten features, comprising triaxial acceleration, triaxial gyroscope, triaxial magnetometer data, and displacement. It enables the recognition of six distinct cattle behaviors: Grazing, Lying-Ruminating, Lying-Resting, Standing-Resting, Walking, and Standing-Ruminating [47]. These behaviors are defined in Table 6.

On the dataset, the original models are divided into two categories, one processing one-dimensional data, such as CNN-1D. Another type requires adding a dimension to the data, such as LeNet-5 AlexNet, MiniVGG-16, ResNet-50, MobileNetV1 [47]. The experimental results of these and S-ResNet models are shown in Table 7. Although LeNet-5, AlexNet, MiniVGG-16, ResNet-50, and MobileNetV1 extracted features in higher dimensions and exhibited FLOPs significantly higher than those of the CNN-1D model, their classification accuracy was lower than that of CNN-1D. This finding suggests that one-dimensional network models are better suited to this dataset. Since this dataset involves six distinct cattle behaviors, its accuracy is slightly lower compared to other studies.

Long Short-Term Memory (LSTM) is a specialized recurrent neural network well-suited for processing sequential data [20]. Bidirectional Long Short-Term Memory (BiLSTM) extends LSTM by introducing a bidirectional processing mechanism [48]. On the dataset, both LSTM and BiLSTM demonstrated superior performance compared to the CNN-1D model, achieving an improvement of approximately 0.7%, while the FLOPs of LSTM were only half that of the CNN-1D model. However, as single-frame data with a time step of 1 was used for classification in this dataset, the advantages of the LSTM network could not be fully utilized.

The S-ResNet 1.0× model achieved the highest classification accuracy of 88.62% (±0.14%), but its FLOPs significantly exceeded those of the CNN-1D and LSTM models. The GNNs-based compression method optimized the S-ResNet model to explore lightweight strategies. By constraining the FLOPs and retaining the framework of the original model, channel pruning was performed to generate three different variants of the S-ResNet model. Among these, S-ResNet 0.1× exhibited extremely low FLOPs of only 0.087M while achieving an accuracy of 87.68% (±0.18%).

**Figure 8:**
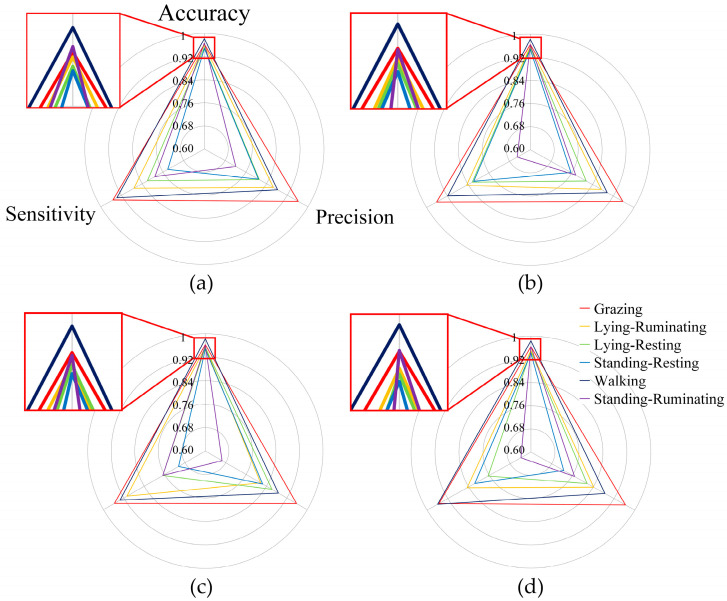
Accuracy, precision, and sensitivity of various behaviors under different S-ResNet model variants. (**a**) S-ResNet 1.0× model; (**b**) S-ResNet 0.75× model; (**c**) S-ResNet 0.4× model; (**d**) S-ResNet 0.1× model.

The accuracy, precision, and sensitivity of six behaviors under different S-ResNet model variants are shown in Figure 8. Among the six behaviors, Walking had the highest accuracy, while Standing-Resting exhibited the lowest accuracy. The accuracy of Lying-Ruminating and Lying-Resting ranked fourth and fifth, respectively, and this rank remained consistent across all S-ResNet model variants. Grazing and Standing-Ruminating ranked second and third in terms of accuracy. In the S-ResNet 1.0× model, the accuracy of Standing-Ruminating was higher than that of Grazing, whereas the opposite was observed in other models. Model pruning had a significant impact on the accuracy of Standing-Ruminating. In terms of precision, Grazing consistently outperformed all other behaviors across all models, while Walking consistently ranked second. The precision of Lying-related behaviors was higher than that of Standing-related behaviors, except the S-ResNet 0.4× model, where Standing-Resting slightly surpassed Lying-Ruminating. In the S-ResNet 1.0×, S-ResNet 0.75×, and S-ResNet 0.4× models, the sensitivity rank was Grazing, Walking, Lying-related behaviors, and Standing-related behaviors. However, in the S-ResNet 0.1× model, the sensitivity of Walking was higher than that of Grazing, and Standing-Resting had a higher sensitivity than Lying-Resting.

The model performed best on Grazing and Walking overall. The model channel pruning was performed under the constraint of overall accuracy to search for the optimal strategy, with slight variations in other evaluation metrics for each behavior category. The pruning process removed channels dependent on specific category features, leading to noticeable changes in the evaluation metrics for those categories. In multi-class classification tasks, predictions across different categories often influence each other, particularly when the categories are more similar, making them more susceptible to the impact of pruning.

### 4.2. Generalization Experiment of Compression Method

To validate the effectiveness of the compression method, the compression method based on GNNs was applied to both MobileNetV1 and MobileNetV2 models. The experiments were conducted using the ImageNet dataset containing 1000 categories [49]. The model compression strategy was conducted with the same settings described in Section 3.1. After completing the strategy search, the optimal strategy was fine-tuned to enhance model accuracy. The model fine-tuning was completed on NVIDIA GeForce RTX 4090. The batch size was set to 128 during fine-tuning, and the training process spanned 200 epochs. The initial learning rate was set to 0.05 and gradually decreased following a cosine annealing schedule as the training progressed.

Table 8 summarizes the accuracy of various models under different FLOPs configurations for other compression methods. For MobileNetV1, the proposed compression method achieved accuracy of 70.82% and 67.04% at FLOPs levels of 328M and 151M, respectively, surpassing the accuracy of models compressed using the baseline and MatePruning methods. Furthermore, when accuracy was similar, the FLOPs of the model obtained by this compression method was 225M, while that of the Uniform 0.75× model was 325M. For MobileNetV2, the proposed method achieved an accuracy 0.37% higher with a 136M FLOPs model compared to the Uniform model with 140M FLOPs. In ResNet-18, under the same FLOPs, the accuracy of the model obtained in this paper is slightly higher than that of the DMCP method.

### 4.3. Comparison with Other Studies

A comparison between this study and related research is shown in Table 9. Studies [8,9,10] share a similar approach to this study, wherein sensors are installed on the neck or legs of cattle to collect data for classification tasks. However, these three studies exclusively utilized wearable devices for data collection without considering the deployment of models to edge devices for real-time inference. The method relies on the combination of a leg-mounted and a neck-mounted accelerometer, requiring two wearable devices to work together [8]. In contrast, our approach achieves comparable classification performance using only a single neck-mounted wearable device with the S-ResNet 1.0× model.

Riaboff et al. utilized a decision tree algorithm with 18 selected features computed in both the time and frequency domains. They combined filters and windows of varying lengths and overlap rates to classify four behaviors: Grazing, Walking, Lying, and Standing [9]. These four behaviors are entirely consistent with the behaviors studied in this article, and the S-ResNet model provides better classification performance than their proposed model. Specifically, under their optimal configuration, the sensitivity for classifying the Walking (94%) surpassed that of the S-ResNet 0.1× model (92.95%) but was lower than S-ResNet 1.0× model (97.56%). For the other three behaviors, their sensitivities (Grazing: 95%; Standing: 82%; Lying: 90%) were all lower than those achieved by the model proposed in this study. Moreover, their overall classification accuracy was 90.25%, which is also lower than S-ResNet model.

In the reference [10], behavior data of dairy cows were recorded using sensor tags placed on the neck. The study classified three behaviors: Grazing, Walking, and Standing, achieving an overall accuracy of 94.35% using a 1D-CNN model. The classification accuracy with the LSTM model was 90.97%, which was lower than the accuracy achieved by the S-ResNet 1.0× model. Their tags can collect radio frequency energy to address power supply issues, whereas the wearable device designed in this study utilizes solar energy to recharge the battery.

The studies in reference [3,4] fundamentally differ from our research. They capture the behavior of dairy cows through fixed devices and train complex deep-learning models to classify these behaviors. Video-based methods outperform sensor-based methods in terms of performance. However, the trade-off is that the computer system must be sufficiently robust to train the models, and the execution time is relatively long. Moreover, the models are too complex to be used for inference on the edge.

To further validate the effectiveness of the proposed method, we selected conventional machine-learning approaches (Random Forest and Decision Tree) and deep models (1D-CNN and LSTM) as baseline methods on the same dataset. These baselines were compared with different variants of the proposed S-ResNet (1.0×, 0.75×, 0.4×, and 0.1×). All methods used identical data preprocessing procedures and the same training/testing split. The comparison results are reported in Table 10. Among the conventional machine-learning methods, Random Forest (91.56% ± 0.10%) outperformed Decision Tree (90.04% ± 0.08%). For the deep-learning baselines, LSTM (92.67% ± 0.11%) achieved slightly higher accuracy than 1D-CNN (92.35% ± 0.07%), indicating that incorporating temporal modeling can improve recognition performance. In contrast, all S-ResNet variants consistently achieved higher accuracy. Under the edge lightweight configuration, S-ResNet-0.1× required only 0.087M FLOPs while still achieving an accuracy of 93.20%, exceeding both 1D-CNN and LSTM. Moreover, its computational cost was reduced by approximately 56.5% compared with LSTM and by approximately 82.0% compared with 1D-CNN, demonstrating the advantage of substantially lowering computation overhead while maintaining high recognition performance.

In summary, compared to other studies, the method proposed in this article can achieve behavior monitoring of freely grazed cattle while ensuring recognition accuracy. By reducing transmission-related power consumption and leveraging solar energy harvesting via solar panels, the proposed system extends device endurance and enables efficient cattle behavior monitoring and management.

## 5. Conclusions

This paper presented an edge-based cattle behavior recognition method that used GNNs for model compression. A wearable device integrating both data acquisition and on-device model inference was developed based on a high-performance embedded microcontroller. A series residual network suitable for single-frame data was designed to reduce the storage of original data, which can be well adapted to data containing time-series information. This paper proposed a compression method based on GNNs, in which the GNNs were used as the fundamental feature extractor within the Actor–Critic architecture. The TD3 algorithm was incorporated to update the reinforcement learning policy. This method enabled the search for an optimal pruning strategy under FLOPs constraints. Additionally, it did not require acceleration by specific hardware architectures, allowing the compressed model to be well-suited for edge devices. Furthermore, the classification model was deployed on an embedded system in this study, which demonstrated the wearable device’s effective inference capability and reduced the power consumption of edge devices.

The S-ResNet 1.0× model achieved a classification accuracy of 94.50% on the collected data for four-class cattle behaviors. It reached an accuracy of 88.62% on the six-class behavior dataset, outperforming the original models on the same dataset. After compression, the S-ResNet 0.1× model attained an accuracy of 93.20% for the four-class classification task and 87.68% for the six-class dataset. Meanwhile, the proposed compression method also demonstrated strong performance when applied to the MobileNetV1, MobileNetV2 and ResNet-18 models. Experimental results showed that, on the wearable device, a single behavior recognition task on the edge consumed only 46.8% of the energy required for cloud-based inference.

This study provides a promising direction for edge-based cattle behavior recognition, yet several challenges remain for deployment in real environments. First, sensor signals may contain outliers due to data-acquisition conditions and individual variability. This calls for incorporating on-device anomaly detection mechanisms, such as adaptive thresholds and sliding-window statistics. Second, long-term wearing on animals is constrained by comfort requirements, and further optimization is still needed in structural design and material selection. Third, real-world conditions such as rain and snow, magnetic interference, and wireless communication blockage may all compromise data stability. Future work will expand the cattle behavior dataset across multiple scenarios, long durations, and different individuals, and will conduct long-term continuous field tests under diverse conditions to facilitate practical deployment in grazing management.

## Figures and Tables

**Table 1:** Definition of four-class cattle behaviors.

Behavior	Description
Grazing	The cattle are searching for grass or eating grass without raising their head.
Lying	The cattle are lying.
Standing	The cattle are standing on all legs.
Walking	The cattle are moving at its normal speed.

**Table 2:** The parameters for model training.

Parameter	Value
The radio of training and test samples	7:3
Learning rate	0.001
Training epochs	100
Batch size	128
Optimizer	Adam
Loss Function	Cross-Entropy Loss

**Table 3:** The parameters for model compression.

Parameter	Value
Batch size	64
Exploration episodes	100
Exploitation episodes	500
Minimum channel retention rate	0.2
Learning rate (Actor)	0.001
Learning rate (Critic)	0.001
Target network soft update coefficient	0.01

**Table 4:** Model inference performance of wearable devices.

Model	MACC	Flash	RAM	Time
S-ResNet 1.0× (STM32Cube.AI compression not selected)	1041.44K	453.16 KiB	17.64 KiB	54 ms
S-ResNet 0.1× (STM32Cube.AI compression not selected)	87.53K	55.73 KiB	8.83 KiB	11 ms
S-ResNet 1.0× (The compression level of STM32Cube.AI is high)	1041.44K	424.43 KiB	17.64 KiB	54 ms

**Table 5:** Comparison of power consumption between edge inference and cloud inference.

Inference Method	Task	Duration	Electric Current	Power Consumption	One-Cycle Power Consumption
Edge inference (real-time inference)	(1) Collect sensor data, GPS positions	30 s	42 mA	0.350 mAh	0.388 mAh
	(2) Inference model on the edge	11 ms	38 mA	1.161 × 10^−4^ mAh	
	(3) Enter sleep mode	270 s	0.5 mA	0.038 mAh	
Cloud inference (latency inference)	(1) Collect sensor data, GPS positions	30 s	42 mA	0.350 mAh	0.829 mAh
	(2) Transmit sensor data and GPS positions to the cloud (3) Inference model in the cloud (4) Receive cattle behavior from the cloud	20 s	80 mA	0.444 mAh	
	(5) Enter sleep mode	250 s	0.5 mA	0.035 mAh	

**Table 6:** Definition of six-class cattle behaviors.

Behavior	Description
Grazing	The cattle are searching for grass or eating grass without raising their heads.
Walking	The cattle are moving at their normal speed.
Lying-Ruminating	The cattle are lying and chewing the food again.
Lying-Resting	The cattle are lying to rest.
Standing-Ruminating	The cattle are standing and chewing the food again.
Standing-Resting	The cattle are standing on all legs to rest.

**Table 7:** Classification results of different models.

Model	FLOPs	Accuracy
LeNet-5	0.586M	86.25% (±0.15%)
AlexNet	81.727M	86.00% (±0.19%)
MiniVGG-16	52.146M	86.77% (±0.20%)
ResNet-50	50.472M	86.04% (±0.15%)
MobileNetV1	23.336M	84.97% (±0.18%)
CNN-1D	0.484M	86.53% (±0.22%)
LSTM	0.200M	87.32% (±0.23%)
BiLSTM	0.531M	87.24% (±0.21%)
S-ResNet 1.0×	1.040M	88.62% (±0.14%)
S-ResNet 0.75×	0.691M	88.49% (±0.16%)
S-ResNet 0.4×	0.416M	87.92% (±0.20%)
S-ResNet 0.1×	0.087M	87.68% (±0.18%)

**Table 8:** Compression results of MobileNetV1, MobileNetV2 and ResNet-18.

Model	Compression Method	FLOPs	Accuracy
MobileNetV1	Uniform 1.0×	569M	70.60%
	Uniform 0.75×	325M	68.45%
	Uniform 0.5×	149M	63.70%
	MatePruning [32]	149M	66.10%
	Ours	328M	70.82%
	Ours	225M	69.26%
	Ours	151M	67.04%
MobileNetV2	Uniform 1.0×	300M	72.30%
	Uniform	140M	67.20%
	Ours	136M	67.57%
ResNet-18	Uniform 1.0×	1.8G	70.10%
	DMCP [50]	1.04G	69.20%
	Ours	1.04G	69.25%

**Table 9:** Comparisons with the related studies.

Reference	Date Acquisition Method	Number of Behavior Types	Classification Method	Accuracy	Energy Harvesting	Edge Inference
[8]	Neck-mounted accelerometer and leg-mounted accelerometer	4	Random Forest	95.10%	No	No
[9]	Colar-mounted accelerometer	4	Decision tree	90.25%	No	No
[10]	Neck-mounted accelerometer	3	1D-CNN and LSTM	94.35%	Yes	No
[3]	Camera (Video-based)	5	EfficientNet-LSTM	97.87%	-	No
[4]	Camera (Video-based)	5	CNN-LSTM	97.60%	-	No
This study	Colar-mounted IMU and GPS	4	S-ResNet 1.0×	94.50%	Yes	Yes
This study	Colar-mounted IMU and GPS	4	S-ResNet 0.1×	93.20%	Yes	Yes

**Table 10:** Comparison results of baseline methods.

Model	FLOPs	Accuracy
Random Forest	—*	91.56% (±0.10%)
Decision tree	—*	90.04% (±0.08%)
1D-CNN	0.484M	92.35% (±0.07%)
LSTM	0.200M	92.67% (±0.11%)
S-ResNet 1.0×	1.040M	94.50% (±0.09%)
S-ResNet 0.75×	0.691M	94.09% (±0.11%)
S-ResNet 0.4×	0.416M	93.53% (±0.10%)
S-ResNet 0.1×	0.087M	93.20% (±0.18%)

* Not available.

## Data Availability

The data presented in this study are available from the corresponding author on reasonable request.
